# Executive Impairment in Alcohol Use Disorder Reflects Structural Changes in Large-Scale Brain Networks: A Joint Independent Component Analysis on Gray-Matter and White-Matter Features

**DOI:** 10.3389/fpsyg.2019.02479

**Published:** 2019-11-26

**Authors:** Chiara Crespi, Caterina Galandra, Marina Manera, Gianpaolo Basso, Paolo Poggi, Nicola Canessa

**Affiliations:** ^1^NEtS Center, Scuola Universitaria Superiore Istituto Universitario di Studi Superiori Pavia, Pavia, Italy; ^2^Cognitive Neuroscience Laboratory, Istituti Clinici Scientifici Maugeri IRCCS, Pavia, Italy; ^3^Psychology Unit, Istituti Clinici Scientifici Maugeri IRCCS, Pavia, Italy; ^4^University of Milano-Bicocca, Milan, Italy; ^5^Radiology Unit, Istituti Clinici Scientifici Maugeri IRCCS, Pavia, Italy

**Keywords:** alcohol use disorder, alcohol chronic consumption, voxel-based morphometry, diffusion tensor MRI, joint independent component analysis, large-scale brain network, rehabilitative applications

## Abstract

Alcohol Use Disorder (AUD) entails chronic effects on brain structure. Neurodegeneration due to alcohol toxicity is a neural signature of executive impairment typically observed in AUD, previously related to both gray-matter volume/density and white-matter abnormalities. Recent studies highlighted the role of meso-cortico-limbic structures supporting the salience and executive networks, in which the extent of neurostructural damage is significantly related to patients’ executive performance. Here we aim to integrate multimodal information on gray-matter and white-matter features with a multivariate data-driven approach (joint Independent Component Analysis, jICA), and to assess the relationship between the extent of damage in the resulting neurostructural superordinate components and executive profile in AUD. Twenty-two AUD patients and 18 matched healthy controls (HC) underwent a Magnetic Resonance Imaging (MRI) protocol, alongside clinical and neuropsychological examinations. We ran jICA on five neurostructural features, including gray-matter density and different diffusion tensor imaging metrics. We extracted 12 Independent Components (ICs) and compared the resulting mixing coefficients in patients vs. HC. Finally, we correlated significant ICs with executive and clinical variables. One out of 12 ICs (IC11) discriminated patients from healthy controls and correlated positively both with executive performance in all subjects, and with lifetime duration of alcohol abuse in patients. In line with previous related evidence, this component involved widespread gray-matter and white-matter patterns including key nodes and fiber tracts of salience, default-mode and central executive networks. These findings highlighted the role of multivariate data integration as a valuable approach revealing superordinate hallmarks of neural changes related to cognition in neurological and psychiatric populations.

## Introduction

Alcohol Use Disorder (AUD) is characterized by prolonged and excessive alcohol consumption, as well as constant concerns about alcoholic drinks despite adverse consequences. This condition can produce relevant alterations at different levels of analysis, from social maladaptation and cognitive impairment (Center for Behavioral Health Statistics and Quality, 2016) to pathological changes affecting anatomo-functional brain regions and networks (De La Monte and Kril, 2014; Fritz et al., 2019). Neuroimaging studies have shown both gray-matter (GM) and white-matter (WM) alterations in AUD (Bühler and Mann, 2011; Yang et al., 2016; Zahr and Pfefferbaum, 2017). Such alcohol-related neurostructural effects involve both decreased GM and WM volume and/or density (Jansen et al., 2015; Xiao et al., 2015; Galandra et al., 2018b), and altered microstructural features (e.g., fractional anisotropy decrease, mean diffusivity increase) in main fiber tracts (Fortier et al., 2014; Chumin et al., 2019). Importantly, neuroimaging studies provided converging evidence about the topological distribution of neurostructural alterations in AUD, showing a diffuse damage pattern that mostly involves fronto-striatal networks alongside frontal WM (De La Monte and Kril, 2014; Suckling and Nestor, 2017). These alterations may represent a neurostructural marker of core cognitive deficits in AUD, including impulsivity and abnormal reward-based choice behavior (see Galandra et al., 2018a). Indeed, major theories proposed to explain cognitive impairment in addiction are related to the dysregulation of either *impulsive vs. reflective* brain systems – the *Control-related deficit theory* (Bechara and Damasio, 2005) – or the *reward vs. stress* systems – the *Reward-related deficit theory* (Koob, 2013). While the latter is more focused on the emotional states associated to craving, seen as a result of the down-regulation of the reward system in favor of the up-regulation of the stress system, the former attributes the emergence of craving to the failure of attention control resources that facilitate impulsive behaviors. Such a view is in line with neuropsychological literature in AUD highlighting the involvement of basic cognitive skills such as memory (Trivedi et al., 2013), processing speed (Sorg et al., 2015), and, more generally, executive functions (Bates et al., 2002; Glass et al., 2009; Le Berre et al., 2017). It is still unknown, however, whether impaired executive profile in AUD reflects a multimodal pattern of neurostructural damage transcending single MRI metrics. Preliminary attempts toward this goal have been pursued by distinct studies relating a global proxy of basic executive functioning (involving psychomotor speed, attention and working memory performances) to the degree of GM atrophy in meso-cortico-limbic structures (Galandra et al., 2018b), and altered functional connectivity in fronto-striatal-limbic networks (Galandra et al., 2019). In line with this evidence, other studies reported an association between attentional/executive deficits and glucose metabolism in the anterior cingulate cortex (ACC) in alcoholics (Goldstein et al., 2004). Altogether, the aforementioned findings consistently suggest that AUD patients’ executive impairment might reflect anatomo-functional alterations involving the *salience network* (SN) (Galandra et al., 2018b, 2019). The latter, indeed, underpins the switch from automatic to controlled effortful processing, associated with the activity of the *default-mode network* (DMN) and *central executive network* (CEN), respectively, when relevant stimuli are detected (Smith et al., 2009; Menon and Uddin, 2010; Goulden et al., 2014).

On this ground, we aimed to integrate multimodal information on GM and WM features in AUD *via* a multivariate data-driven approach – joint Independent Component Analysis (jICA) – suitable to identify superordinate patterns at the *network level* (Calhoun, 2018). While this method has been successfully applied to other neurological and psychiatric conditions (Guo et al., 2012; Sui et al., 2013; Teipel et al., 2014; Kim et al., 2015), to the best of our knowledge no previous research has taken a comparable multivariate approach to investigate the ICs discriminating AUD patients from healthy controls (HC), and to assess their relationship with a superordinate proxy of impaired executive profile.

We expected to reconcile separate single-modality findings (Galandra et al., 2018b, 2019) into a unique consistent framework in which the ICs differentiating AUD patients from HC reflect neurostructural alterations of nodes and connections involving the salience network, with their mixing coefficient reflecting the degree of patients’ executive impairment.

## Materials and Methods

### Participants

Twenty-two AUD patients and 18 HC took part in the study, including a semi-structured interview about alcohol and nicotine use habits, a neuropsychological assessment, and a multimodal Magnetic Resonance Imaging (MRI) session. AUD patients were consecutively enrolled from the Functional Rehabilitation Unit of ICS Maugeri-Pavia (Italy), while HC were recruited *via* local advertisement. HC were matched for age and education to AUD patients, and groups were also balanced for gender (see Table 1 for details).

**Table 1:** Demographics and clinical information about substance habits.

	AUD (*n* = 22)	HC (*n* = 18)	*p*
Age (years; mean ± SD)	45.56 ± 7.99	45.11 ± 8.69	0.426
Education (years; mean ± SD)	9.91 ± 2.65	10.11 ± 2.78	0.405
Gender (m:f)	13:9	11:7	0.890
Smoking status (yes:no)	18/4	6/12	0.184
Duration of alcohol use (years; range, mean ± SD)	1–26 (10.11 ± 6.56)	—	—
Average daily alcohol dose (UA; range, mean ± SD)	5–32 (14.34 ± 6.66)	—	—

*AUD, AUD patients; HC, healthy controls; Daily UA, units of alcohol*.

*The table reports demographic data related to age, education, gender and smoking status of AUD patients and healthy controls, alongside clinical information about alcohol use history and daily dose, and nicotine consumption, of AUD patients*.

Inclusion criteria for AUD subjects were age between 20 and 60 years and a diagnosis of alcohol dependence according to DSM-V criteria. We excluded HC in the presence and/or history of alcohol abuse. Exclusion criteria for both groups were the presence and/or history of neurological/psychiatric disorders other than AUDs, or any comorbid disorder except for smoking dependence, family history of neurological/psychiatric disorders, major medical disorders (e.g., kidney or liver diseases, severe diabetes and/or malnutrition), current use of any psychotropic substance/medication, past brain injury or loss of consciousness, inability to complete the neuropsychological assessment, and presence of contraindications to MRI.

AUD patients were enrolled after being detoxified for at least 10 days by means of medically supported standard treatments. However, they underwent MRI protocol only after at least 8 days without benzodiazepine treatment. HC were asked to be abstinent at least 10 days before the scanning day. We ascertained the abstinence of HC *via* a semi-structured interview about the consumption of alcoholic drinks covering that time. None of the participants received financial incentives to join the research protocol. Each enrolled subject had signed informed consent to the experimental protocol, which was approved by the Ethical Committee of ICS Maugeri (Pavia, Italy). The investigation was conducted in accordance with the latest version of the Declaration of Helsinki.

### Clinical Interview, Neuropsychological Evaluation, and Data Analysis

AUD patients underwent a semi-structured interview conducted by an expert clinician about their drinking history, including the type, the amount, and lifetime duration of alcohol use. We used the average number of standard units of alcohol (UA) per day (1 UA = 330 ml beer, 125 ml wine, or 40 ml hard liquor = 12 g of ethanol) as a proxy of alcohol consumption (Table 1).

Neuropsychological assessment was performed using the Brief neuropsychological examination (ENB-2, Mondini, 2011), encompassing 15 tests assessing attention (trail making test, i.e., TMT-A and TMT-B), verbal short-term (digit span) and long-term memory (immediate and delayed prose memory), working memory (10″ and 30″ interference memory), executive functions (TMT-B, cognitive estimation, abstract reasoning, phonemic fluency, clock drawing, overlapping pictures), perceptive and praxis skills (praxis abilities, spontaneous drawing, copy drawing task). The ENB returns a global score, as well as different sub-scores for each task. The analysis of the resulting neuropsychological data has been previously described by Galandra and colleagues (Galandra et al., 2018b, 2019) and reported in Supplementary Table S1.

### Magnetic Resonance Imaging Protocol and Data Acquisition

We use a 3 Tesla General Electrics Discovery MR750 scanner (GE Healthcare), equipped with a 16-channel phased array head coil, to run a multimodal MRI protocol including (1) a high-resolution 3D T1-weighted IR-prepared FSPGR (BRAVO) brain scan acquired along the AC-PC plane (152 slices, FOV = 24 cm, reconstruction matrix = 256 × 256, slice thickness = 1 mm); (2) a diffusion tensor imaging (DTI) scan based on a single-shot echo planar sequence (TR/TE = 8,986/80; FOV = 256 mm2; 56 sections; bandwidth = 250.0, 2 mm isotropic resolution), with diffusion gradients applied along 81 non-collinear directions (*b* = 1,000 s/mm^2^), plus two non-diffusion weighted volumes. We also collected a T2-weighted image in order to explore any possible accidental diagnosis.

### Voxel-Based Morphometry Data Pre-Processing

The pre-processing and statistical analysis of T1-weighted anatomical data were based on SPM12^1^ and the CAT12 toolbox^2^. Pre-processing included bias-field inhomogeneities correction; spatial normalization using the DARTEL algorithm (Ashburner, 2007); and segmentation into GM, WM, and cerebrospinal fluid (CFS) (Ashburner and Friston, 2005). We did not apply the Jacobian modulation of segmented GM images, which corrects for volume change during spatial normalization, since this procedure has been shown to decrease the sensitivity to morphometric abnormalities (Radua et al., 2014). Our results thus involve GM density, i.e., GM volume relative to WM and CSF volume. Finally, a smoothing kernel of 8 mm (FWHM) was applied to the normalized segmented GM images. The resulting smoothed normalized GM images were fed into joint ICA.

### DTI Data Pre-Processing

We performed the pre-processing of DTI data with the FMRIB Software Library tools (FSL; http://fsl.fmrib.ox.ac.uk/fsl/fslwiki/). Single-subject datasets were first corrected for eddy current distortions and motion artifacts, skull-stripped, and finally, as a result of the fitting of the diffusion tensor model at each voxel, maps of diffusion scalar indices were generated. We then carried out DTI group analyses with Tract-Based Spatial Statistics (TBSS) (Smith et al., 2006), involving a voxelwise non-linear registration of all participants’ fractional anisotropy (FA) maps that, once aligned, are affine-transformed on a standard space (1 mm × 1 mm × 1 mm MNI152). After co-registration, FA maps are averaged to create a mean FA image, and then used to generate a mean FA tract skeleton, representing all common tracts across subjects. We applied to the mean FA skeleton image a threshold of 0.20 to exclude from further analyses those parts of the skeleton that could not ensure a good correspondence across subjects. Finally, to account for residual misalignments after the initial non-linear registration, all subjects’ FA data were projected onto the thresholded mean FA skeleton, creating a 4D dataset of all subjects’ FA skeletonized data. In addition, we ran the non-FA TBSS script on maps of mean (MD), radial (RD), and axial (AD) diffusivities. The resulting skeletonized data were then fed into joint ICA.

### Joint Independent Component Analysis

We used jICA – a multivariate approach integrating data from different neuroimaging modalities (i.e., features) unveiling covariance patterns of signal change across the brain – to estimate maximally independent components (ICs) at the topological level (i.e., spatial maps) for single features, which are then combined by a shared loading (mixing) parameter (Calhoun et al., 2006).

Shared information from GM density and DTI-invariant skeletonized (i.e., FA, AD, MD, RD) images were obtained using the Fusion ICA Toolbox (FIT v2.0c; http://icatb.sourceforge.net). Specific methodological details on this approach have been already described (Calhoun et al., 2006). Briefly, each participant’s GM and DTI-invariant skeletonized images were first separately converted into a one-dimensional row vector. The initial data matrix was thus formed by arraying 22 GM, FA, AD, MD, and RD vectors of AUD patients and 18 GM, FA, AD, MD, and RD vectors of HC into a 40-row subjects-by-voxels matrix. Each feature dataset was then combined into a single data (participant × feature) matrix. All feature maps were normalized, resulting in the same average sum-of-square (computed across all voxels and subjects for each modality) and thus in equal data ranges. We used standard PCA to reduce the dimensionality of the data to 12 ICs, with this value being estimated for each feature using the minimum description length (MDL) criterion (Li et al., 2007). The Infomax algorithm (Bell and Sejnowski, 1995) was then used to decompose the reduced feature-matrix to maximally independent component images and subject-specific mixing (loading) coefficients. This jICA approach was repeated 50 times in Icasso^3^. The resulting 12 ICs were clustered to ensure the consistency and reliability of the decomposition, which are quantified using a quality index (QI) ranging from 0 to 1, reflecting the difference between intra-cluster and extra-cluster similarity (Himberg et al., 2004).

Mixing coefficients, reflecting how strongly each participant contributes to the relationship described in a given IC, were fed into a two-sample *t*-test assessing a significant difference between AUD patients and HC. Only significant components reflecting neurostructural changes in AUD patients (i.e., mixing coefficients AUD > mixing coefficients HC) were considered in subsequent analyses.

On this basis, we aimed to investigate whether ICs differentiating patients from HC additionally confirmed the involvement of *salience network* regions as neurostructural markers of the neuro-cognitive impairment associated with AUD (Galandra et al., 2018b, 2019). To this purpose, we finally correlated mixing coefficients with (1) a measure of each participant’s executive profile, obtained *via* a multivariate data reduction approach (see Galandra et al., 2018b for detailed information about the statistical procedure) and (2) clinical variables (i.e., lifetime duration of alcohol abuse and daily alcohol consumption) in AUD patients.

The anatomical localization of significant clusters was performed with the JHU White-Matter Tractography Atlas and the JHU ICBM-DTI-81 White-Matter Labels (Wakana et al., 2007; Hua et al., 2008) for DTI features, while the SPM Anatomy toolbox (Eickhoff et al., 2005) was used to localize gray-matter features.

## Results

### Joint Components of Neurostructural Change in Alcohol Use Disorder Patients Vs. Healthy Controls

**Figure 1:**
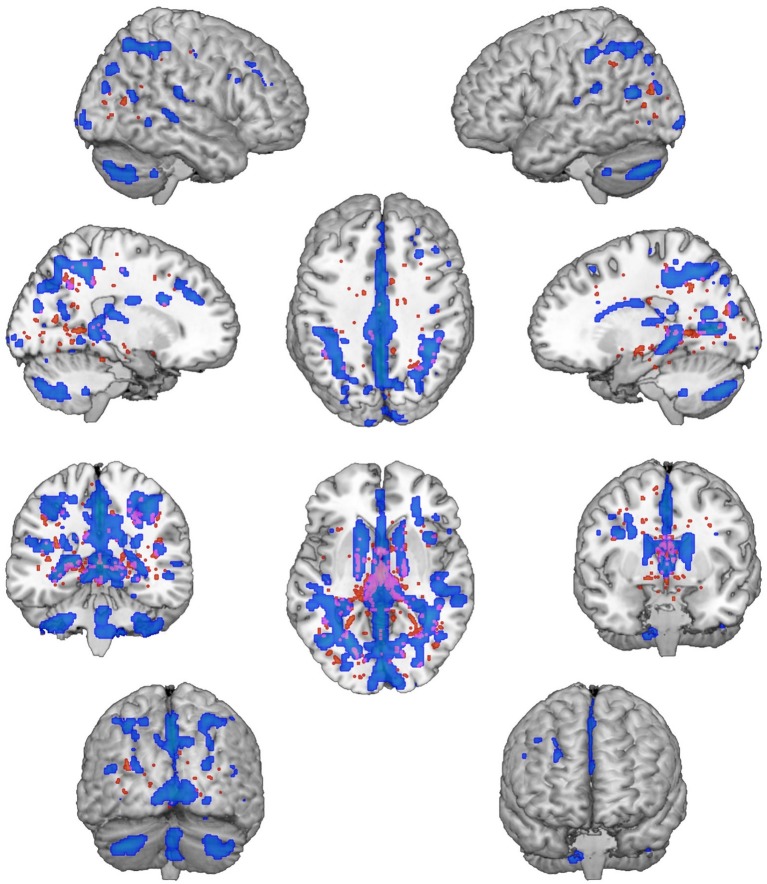
IC06 Pattern. The figure depicts the covariance pattern of IC06 emerging from joint ICA. Statistical maps are thresholded at *z* = 2.5 for visualization purposes. Gray-matter clusters and white-matter clusters including all DTI metrics (i.e., FA, MD, AD, RD) are shown in blue-green and red-yellow colors, respectively.

**Figure 2:**
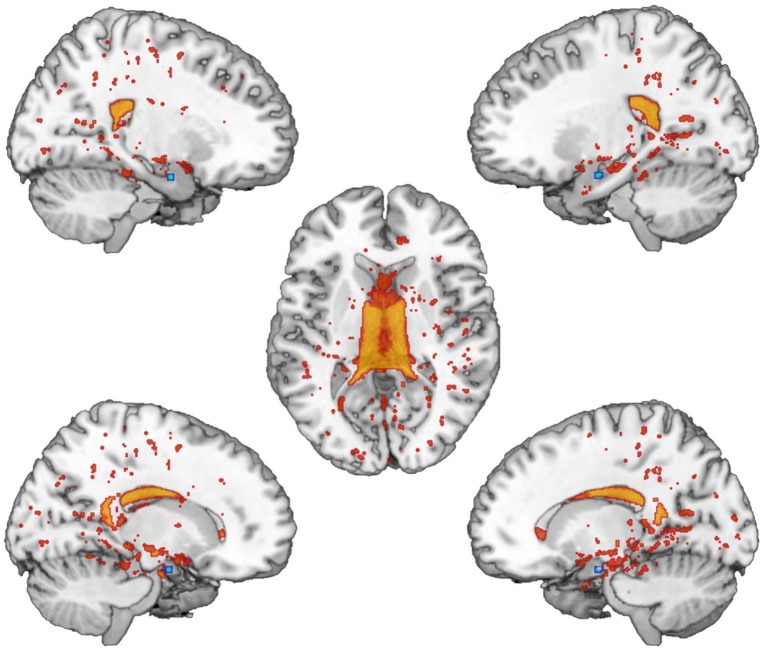
IC08 Pattern. The figure depicts the covariance pattern of IC08 resulting from joint ICA. Statistical maps are thresholded at *z* = 2.5 for visualization purposes. Gray-matter clusters and white-matter clusters including all DTI metrics (i.e., FA, MD, AD, RD) are shown in blue-green and red-yellow colors, respectively.

**Figure 3:**
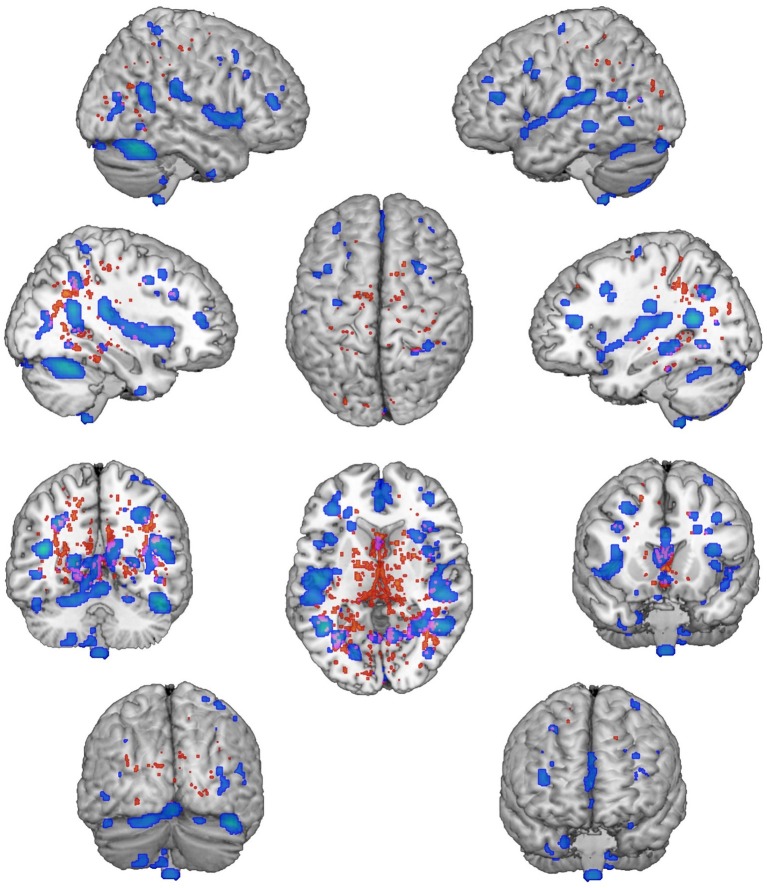
IC11 Pattern. The figure depicts the covariance pattern of IC11 highlighted by joint ICA. Statistical maps are thresholded at *z* = 2.5 for visualization purposes. Gray-matter clusters and white-matter clusters including all DTI metrics (i.e., FA, MD, AD, RD) are shown in blue-green and red-yellow colors, respectively.

We found significantly different mixing coefficients, in AUD patients vs. HC, in three out of 12 joint ICs (IC06, IC08, IC11), with *p* = 0.004 as adjusted significance threshold applied to control for multiple comparisons (Bonferroni correction). All these ICs were associated with a quality index >0.95, indicating a highly stable ICA decomposition. While mixing coefficients of IC06 and IC11 were higher in patients compared with controls [IC06: *t*(38) = −5.69, *p* < 0.001; IC11: *t*(38) = −3.82, *p* < 0.001], IC08 displayed the opposite pattern [IC08: *t*(38) = 5.17, *p* < 0.001]. IC06 involved a widespread GM pattern encompassing the sensorimotor cortex and supplementary motor area, cingulate cortex, and precuneus, subcortical nuclei (bilateral thalamus, left caudate), plus an extensive sector of the occipital cortex (calcarine cortex, cuneus, lingual gyrus), and bilateral cerebellum (crus II). The distribution of DTI indices for this component involved commissural (body of corpus callosum and forceps major), projections (bilateral anterior thalamic radiation and superior corona radiata, as well as the right cerebral peduncle), and associative (fornix plus stria terminalis, as well as the posterior sectors of bilateral superior and inferior longitudinal fasciculi, inferior fronto-occipital fasciculus and cingulum bundle) fibers (Figure 1). IC08 was represented by a subcortical GM pattern including bilateral amygdala and left hippocampus, and by a widespread DTI pattern primarily involving all sectors of corpus callosum (genu, body, and splenium), forceps minor and forceps major, anterior thalamic radiations and cerebral peduncles, the anterior limb of internal capsule and the fornix (body and column) (Figure 2). IC11 involved bilaterally the middle frontal gyrus, insula, anterior and posterior sectors of the cingulate cortex, distinct sectors of the temporal (superior and middle temporal gyri, supramarginal gyrus), parietal (precuneus, angular gyrus) and occipital (lingual and fusiform gyri) lobes, plus the left hippocampus and the cerebellum (crus I). Here, the overall DTI pattern encompassed commissural (genu and body of corpus callosum, forceps major and forceps minor), projection (anterior limb of internal capsule and thalamic radiations, as well as superior corona radiata), and associative (both fornix body and column, along with superior and inferior longitudinal fasciculi, inferior fronto-occipital fasciculus and cingulum bundle, with a right hemispheric prevalence) fibers (Figure 3). Detailed information about localization of significant ICs is reported in Supplementary Tables S2–S4.

### Relationship Between Independent Components and Executive/Clinical Variables

Among the three ICs differentiating AUD patients from HC, only IC6 and IC11 mixing coefficients presented a difference pattern (i.e., AUD > HC) suggesting a stronger contribution of signal covariance in patients than HC, and thus reflecting a possible neurostructural alteration characterizing AUD. However, the IC11, but not IC06 mixing coefficients (*r* = −0.23, *p* = 0.159), were significantly correlated with participants’ executive profile (*r* = −0.54, *p* < 0.001).

Correlation analyses in the patient group highlighted a significant positive correlation between lifetime duration of alcohol abuse and IC11 (*r* = 0.51, *p* = 0.016), but not with IC6 mixing coefficients (*r* = 0.37, *p* = 0.09). No significant correlations with daily alcohol consumption was observed neither in IC6 (*r* = −0.002, *p* = 0.994) nor in IC11 mixing coefficients (*r* = −0.05, *p* = 0.499). Scatterplots of all correlations are reported in Supplementary Figures S1–S6.

## Discussion

We used jICA to investigate supramodal patterns of covariance (ICs) reflecting shared information across several neurostructural features including GM density and distinct WM microstructural properties. We then explored the relationship between the ICs discriminating AUD patients from HC and the overall executive profile highlighted by a multivariate analysis of performance in several neuropsychological tasks (Galandra et al., 2018b). This approach aimed to investigate the connection between a superordinate proxy of AUD patients’ cognitive impairment transcending single tasks, and spatial maps integrating multimodal MRI information on the underlying neuro-anatomical alterations. As far as we know, this is the first research *combining* two parallel data-driven multivariate analyses of neurostructural and behavioral data, and their relationship, in AUD. The resulting evidence confirmed previous reports, from univariate analyses of single MRI modalities, of a defective interplay, in AUD, between large-scale brain networks underlying the salience-based switch from automatic to controlled cognition and behavior (e.g., Galandra et al., 2018b, 2019).

First, three out of the 12 extracted ICs (IC06, IC08, IC11) differentiated AUD patients from HC. The mixing coefficients of IC06 and IC11 were significantly higher in AUD patients compared to controls, while we found the opposite pattern for IC08. Higher mixing coefficients in a given IC are suggestive of a greater contribution to the original features by its constituting regions (Calhoun et al., 2006; Kim et al., 2015). The present evidence seems thus to indicate that the regions included in IC06 and IC11 are more tightly related to neural changes associated with chronic alcohol consumption than IC08. Importantly, IC11 mixing coefficients were also significantly related to executive performance in the whole sample, and to lifetime duration of alcohol abuse in AUD patients.

This component encompassed a set of GM regions including the insula bilaterally and both the anterior (ACC) and posterior (PCC) cingulate cortex, alongside commissural (corpus callosum) and major associative fiber tracts (superior and inferior longitudinal fasciculi, inferior fronto-occipital fasciculus and cingulum bundle). The fronto-insular cortex and the anterior cingulate cortex (ACC) are two interconnected key components of the *salience network* (SN), typically co-activated by behaviorally relevant stimuli (Seeley et al., 2007; Goulden et al., 2014). Both these regions are connected with sensory and motor areas, and their activation is considered to underpin the switch between *default-mode* and *central executive networks* (Goulden et al., 2014). Such a general-purpose function fits with the insula role as a site of multimodal convergence of signals concerning sensory and affective processing (Uddin et al., 2014), likely supporting salience-related top-down mechanisms such as impulse control and self-regulation (see Sullivan et al., 2013). Therefore, it is not surprising to observe neurostructural alterations in a portion of the insula previously associated with abnormal network efficiency and functional connectivity (Wang et al., 2018), hypo-connectivity with precuneus, SMA, postcentral, lingual/vermis, and fusiform gyri (Vergara et al., 2017), and a perfusion deficit (Sullivan et al., 2013), in AUD patients. The possible relationship between such insular dysfunction and a defective interplay among salience, default mode, and executive control networks in AUD (Sullivan et al., 2013) is indirectly supported by the present evidence of co-occurrent structural and functional alterations in the ACC (Müller-Oehring et al., 2015; Galandra et al., 2018b). The latter is a key node of the reward pathway (Haber and Knutson, 2010), in which neural mechanisms of performance monitoring signal to the fronto-parietal executive network the need of behavioral adjustments (Ridderinkhof et al., 2004). Thus the ACC is a well-suited region to promote salience-based behavioral adaptations, which require to switch from default to controlled processing. Therefore, the impairment of both these networks in AUD (Chanraud et al., 2011; Sullivan et al., 2013; Galandra et al., 2018b, 2019) fits with previous evidence of a connection between altered ACC activity and craving, likely promoting relapses (Koob and Le Moal, 2008). Further evidence of an altered interplay between salience and default mode networks in AUD is represented by IC11 including the posterior cingulate cortex, a key node of default mode network (Greicius et al., 2009) in which decreased coherence of the spontaneous BOLD fluctuations has long been known as a neural marker of impaired functional connectivity (Chanraud et al., 2011).

Several evidences support the relationship between such alterations and impaired executive functioning. On the one hand, GM atrophy in the insular and anterior cingulate cortex predicts executive deficits, mainly involving attention and working-memory, in AUD patients (Galandra et al., 2018b). Moreover, abstinence seems to reverse alcohol-related morphological alterations in these regions (Fritz et al., 2019) and restore connectivity within and between the salience and executive networks (Kohno et al., 2017), with these changes paralleling an improvement of executive skills (Le Berre et al., 2017).

While the present findings support previous data on the relationship between AUD patients’ executive impairment and GM nodes within the salience network, our analytic approach allowed to extend this evidence to WM connections. In particular, the corpus callosum (genu) and the cingulum bundle included in IC11 connect the key nodes of large-scale functional networks (Van Den Heuvel et al., 2009) in which functional alterations have been ascribed both to GM loss in crucial nodes, and to macro- and/or micro-structural impairments in WM tracts connecting them (Peer et al., 2017). It is noteworthy that the genual fibers interconnect homologous prefrontal regions such as the dorsolateral prefrontal cortex (DLPFC) (Voineskos et al., 2010) – an important hub of the central executive network (e.g., Seeley et al., 2007; Chen et al., 2013; Marstaller et al., 2015) – and the ACC (van der Knaap and van der Ham, 2011), and that both the genu microstructure and the DLPFC function have been associated to executive performance (Zahr et al., 2009). All these data converge to suggest that the well-established damage of genual fibers in AUD (Pfefferbaum et al., 2006), *via* DLPFC dysfunction, can decrease the efficiency of the central executive network.

A limitation of our work concerns the small-to-moderate sample size, due to the strictness of inclusion criteria and the accurate control of possible nuisance variables, which highlights the need of confirmatory studies before strong conclusions can be drawn on the multimodal neural bases of executive deficits in AUD. Moreover, the lack of information about brain activity limits our conclusions to the neurostructural level, thus preventing inferences about possible consequences of structural damage in terms of impaired functional connectivity and/or compensatory mechanisms. Future studies might fill these gaps by addressing a more comprehensive view integrating functional and structural connectivity measures in larger samples. Importantly, however, the present results pave the way for such further investigations by starting to unveil the relationship among cognitive impairment in AUD and the topographic properties of multimodal “neurostructural” ICs differentiating AUD patients from HC. Building on the present evidence, longitudinal studies might also benefit from the application of multivariate analytic approaches to explore multimodal changes and their association with cognitive status, in relation to abstinence and relapses, or as a result of rehabilitative interventions.

In conclusion, the present findings confirm and integrate into a coherent framework previously scattered evidence about the involvement of key nodes of salience, default mode, and central executive networks, and their structural connections, as reliable neuroimaging markers of executive impairment in AUD. The relevance of IC11 in discriminating AUD patients from HC and its uniqueness in synthesizing different facets of the neurostructural damage in AUD are further supported by the positive relationship between its associated GM and WM patterns and lifetime duration of alcohol abuse. From the methodological standpoint, the present data confirm the consistency between the outputs of multivariate jICA and standard univariate analyses (see Galandra et al., 2018b), thus supporting the notion that jICA can capture the complexity of the neurostructural impairment in AUD based on unique coefficients expressing covariance patterns of morphometric GM and microstructural WM data.

## Data Availability Statement

The datasets generated for this study are available on request to the corresponding author.

## Ethics Statement

The studies involving human participants were reviewed and approved by the Ethics Committee of IRCCS ICS Maugeri, 27100, Pavia, Italy. The patients/participants provided their written informed consent to participate in this study.

## Author Contributions

CC, CG, and NC participated in study design and conceptualization and in manuscript drafting and revising. CG, MM, GB, PP, and NC collected data. CC and CG performed data analysis and interpretation. CC, CG, MM, GB, PP, and NC approved the final version of the manuscript.

### Conflict of Interest

The authors declare that the research was conducted in the absence of any commercial or financial relationships that could be construed as a potential conflict of interest.

## Footnotes

^1^http://www.fil.ion.ucl.ac.uk/spm

^2^http://www.neuro.uni-jena.de/cat/

^3^http://research.ics.aalto.fi/ica/icasso/
