# Supplementary material for: Executive Impairment in Alcohol Use Disorder Reflects Structural Changes in Large-Scale Brain Networks: A Joint Independent Component Analysis on Gray-Matter and White-Matter Features
Source: Front Psychol. 2019 Nov 26;10:2479. doi: 10.3389/fpsyg.2019.02479 (PMC6988803; doi:10.3389/fpsyg.2019.02479)
Supplement: Supplementary file 1 [file Data_Sheet_1.docx]

**SUPPLEMENTARY MATERIALS**

**Executive impairment in Alcohol Use Disorder reflects structural changes in large-scale brain networks: A joint Independent Component Analysis on grey-matter and white-matter features**

by Crespi, Galandra et al.

**Table S1. Neurocognitive performance**

|  | HC  (mean ± sd) | AUD  (mean ± sd) | DoF | T/U* | p-value | FDR |
| --- | --- | --- | --- | --- | --- | --- |
| ENB-2 Global Score | 84.11 ± 7.39 | 77.68 ± 8.21 | 38 | 2.575 | 0.007 | 0.021 |
| Digit Span | 5.78 ± 1.17 | 5.73 ± 1.24 | 38 | 184.0* | 0.358 | 0.420 |
| Immediate recall | 15.22 ± 4.57 | 12.82 ± 4.37 | 38 | 1.695 | 0.049 | 0.110 |
| Delayed recall | 20.33 ± 5.04 | 18.68 ± 5.20 | 38 | 1.013 | 0.159 | 0.318 |
| Interference memory 10’’ | 7.61 ± 1.61 | 6.23 ± 1.90 | 38 | 105.0* | 0.005 | 0.018 |
| Interference memory 30’’ | 6.94 ± 2.01 | 6.41 ± 2.22 | 38 | 169.5* | 0.221 | 0.362 |
| TMT-A | 19.16 ± 5.45 | 29.05 ± 5.98 | 38 | -5.408 | < 0.001 | < 0.001 |
| TMT-B | 68.56 ± 21.79 | 84.91 ± 35.28 | 38 | 132.5* | 0.044 | 0.110 |
| Token Test | 4.97 ± 0.12 | 4.93 ± 0.18 | 38 | 182.0* | 0.338 | 0.420 |
| Phonemic Fluency | 12.72 ± 3.08 | 12.42 ± 3.28 | 38 | 0.296 | 0.384 | 0.420 |
| Abstract verbal reasoning | 5.67 ± 0.97 | 5.59 ± 0.85 | 38 | 185.5* | 0.368 | 0.420 |
| Cognitive estimation | 4.72 ± 0.46 | 4.73 ± 0.55 | 38 | 190.5* | 0.420 | 0.420 |
| Overlapping figures | 36.94 ± 5.51 | 31.09 ± 5.72 | 38 | 3.271 | 0.001 | 0.006 |
| Copy drawing | 1.83 ± 0.38 | 1.64 ± 0.58 | 38 | 166.5* | 0.198 | 0.356 |
| Spontaneous Drawing | 1.89 ± 0.32 | 1.73 ± 0.55 | 38 | 174.0* | 0.263 | 0.395 |
| Clock Test | 9.39 ± 2.35 | 8.36 ± 2.45 | 38 | 102.5* | 0.004 | 0.018 |
| Praxic Abilities | 6.00 ± 0.00 | 5.95 ± 0.21 | 38 | 189.0* | 0.409 | 0.420 |

The table reports information about both global and single task scores (mean, standard deviation) related to neurocognitive performance obtained at the Brief Neuropsychological Exam-2 (ENB-2, Mondini et al. 2011) by AUD patients and healthy controls. We additionally reported details about statistics used for group comparisons and the relative results. (*) indicates results from non-parametric tests (Mann-Whitney U) used to investigate group comparisons in case of non-normally distributed data. In all the other cases, we assessed group differences with the Student’s t-test for independent samples.

In previous studies (Galandra et al., 2018b, Galandra et al., 2019) we showed that neurostructural and neurofunctional alterations in AUD patients correlate with a proxy of executive performance, obtained as a result of a data reduction procedure (i.e., Principal Component Analysis) applied on ENB-2 scores. Factorial scores of the principal component #3 including TMT-A, Interference memory 10” and 30” tasks (underlined in the table) represent such a proxy, reflecting the basic executive profile we then correlated with ICs emerging from joint ICA. *AUD=AUD patients; HC=healthy controls; DoF=Degrees of Freedom; T=Student’s t-Test; U=Mann-Whitney U Test; FDR=False Discovery Rate adjustment applied on raw p-values.*

**Table S2. Localization and coordinates of IC6**

| **Grey Matter** | | | | | | |
| --- | --- | --- | --- | --- | --- | --- |
| *H* | *Region* | *k* | *Z* | *x* | *y* | *z* |
| R | MCC | 7740 | 6.52 | 2 | -15 | 51 |
| L | Precuneus |  | 5.92 | 0 | -48 | 45 |
| L | Calcarine Gyrus |  | 5.47 | 2 | -86 | 2 |
| L | Cuneus |  | 5.23 | 0 | -72 | 32 |
| L | Posterior-Medial Frontal |  | 5.10 | 0 | 8 | 46 |
| L | PCC |  | 4.20 | 0 | -54 | 33 |
| R | Calcarine Gyrus |  | 4.08 | 10 | -94 | -3 |
| R | Precuneus |  | 3.68 | 14 | -68 | 28 |
| L | Thalamus | 2668 | 8.46 | 0 | -14 | 6 |
| R | Hippocampus |  | 3.71 | 32 | -38 | 2 |
| R | Thalamus |  | 3.67 | 9 | -22 | 18 |
| R | IPL | 1045 | 4.97 | 38 | -46 | 51 |
| R | SPL |  | 3.99 | 28 | -66 | 56 |
| R | Postcentral Gyrus |  | 3.08 | 45 | -36 | 56 |
| L | IPL | 957 | 4.33 | -33 | -54 | 51 |
| L | Postcentral |  | 3.90 | -39 | -36 | 45 |
| L | SPL |  | 3.04 | -20 | -70 | 54 |
| L | Caudate Nucleus | 751 | 4.21 | -15 | 15 | 48 |
| L | Cerebelum (Crus 2) | 648 | 4.55 | -33 | -75 | -40 |
| R | Cerebelum (Crus 2) | 579 | 3.68 | 32 | -80 | -38 |
| R | Cerebelum (IX) | 543 | 3.67 | 15 | -54 | -50 |
| L | Hippocampus | 440 | 4.92 | -33 | -36 | -4 |
| L | Middle Temporal Gyrus | 256 | 4.90 | -40 | -64 | 18 |
| R | Rolandic Operculum | 250 | 3.71 | 46 | -26 | 16 |
| R | Heschls Gyrus |  | 3.51 | 44 | -22 | 15 |
| R | SupraMarginal Gyrus |  | 2.97 | 58 | -26 | 20 |
| R | Middle Frontal Gyrus | 160 | 3.58 | 27 | 34 | 30 |
| R | IFG (p. Opercularis) | 122 | 4.15 | 38 | 15 | 27 |
| R | Middle Temporal Gyrus | 118 | 3.23 | 60 | -30 | 0 |
| L | Superior Medial Gyrus | 92 | 3.50 | -2 | 48 | 18 |
| **White Matter** | | | | | | |
| *H* | *Metric and Region* | *k* | *Z* | *x* | *y* | *z* |
| - | FA - Fornix | 237 | 7.65 | 0 | -4 | 16 |
| R | FA - Anterior Thalamic Radiation | 41 | 4.16 | 9 | -29 | 15 |
| R | FA - Superior Corona Radiata | 40 | 3.9 | 18 | -9 | 38 |
| R | FA - Corpus Callosum (body) | 38 | 3.55 | 7 | -17 | 27 |
| L | FA - Corpus Callosum (body) | 38 | 3.89 | -5 | -16 | 26 |
| L | FA - Superior Corona Radiata | 36 | 3.33 | -17 | -10 | 37 |
| L | FA - Anterior Thalamic Radiation | 35 | 3.80 | -15 | -32 | 11 |
| R | FA - Corpus Callosum (splenium) | 33 | 3.93 | 27 | -52 | 3 |
| L | FA - Cingulum (hippocampus) | 30 | 3.54 | -23 | -49 | 3 |
| L | FA - Inferior Fronto-occipital Fasciculus | 28 | 5.05 | -33 | -80 | 20 |
| L | FA - Inferior Fronto-occipital Fasciculus | 25 | 3.45 | -35 | -10 | 20 |
| L | FA - Inferior Fronto-occipital Fasciculus | 20 | 4.89 | -13 | -78 | 1 |
| R | FA - Superior Longitudinal Fasciculus | 19 | 4.15 | 43 | -67 | 14 |
| R | FA - Anterior Thalamic Radiation | 16 | 3.98 | 1 | -18 | -7 |
| L | FA - Superior Longitudinal Fasciculus | 15 | 4.13 | -42 | -52 | 39 |
| L | FA - Anterior Thalamic Radiation | 15 | 3.43 | -7 | -21 | 14 |
| L | FA - Anterior Thalamic Radiation | 14 | 4.00 | -4 | -15 | 13 |
| R | FA - Superior Corona Radiata | 14 | 2.84 | 19 | -24 | 37 |
| L | FA - Inferior Fronto-occipital Fasciculus | 13 | 3.60 | -20 | -72 | 1 |
| L | FA - Inferior Fronto-occipital Fasciculus | 12 | 3.29 | -40 | -74 | 9 |
| L | FA - Anterior Thalamic Radiation | 11 | 3.57 | -23 | -34 | 5 |
| R | FA - Superior Longitudinal Fasciculus | 10 | 3.51 | 30 | -56 | 38 |
| R | FA - Superior Longitudinal Fasciculus | 10 | 3.68 | 41 | -55 | 43 |
| L | RD - Corpus Callosum (body) | 10 | 4.33 | -2 | 6 | 24 |

The table lists the localization of *local maxima* of significant clusters encompassed in IC6. We reported clusters with size ⪰ 80 voxels for grey matter, and ⪰10 voxels for white matter microstructural features. The anatomical localization of significant clusters was performed with the JHU White-Matter Tractography Atlas and the JHU ICBM-DTI-81 White-Matter Labels (Hua et al., 2008; Wakana et al., 2007) for DTI features, while the SPM Anatomy toolbox (Eickhoff et al., 2005) was used to localize grey matter features. *H=hemisphere; k=cluster size (i.e., number of voxels); Z=z-scores; x, y, z=MNI coordinates; MCC=Middle Cingulate Cortex; PCC=Posterior Cingulate Cortex; IPL=Inferior Parietal Lobule; SPL= Superior Parietal Lobule; IFG=Inferior Frontal Gyrus; FA=Fractional Anisotropy; AD=Axial Diffusivity; MD=Mean Diffusivity; RD=Radial Diffusivity.*

**Table S3. Localization and coordinates of IC8**

| **Grey Matter** | | | | | | |
| --- | --- | --- | --- | --- | --- | --- |
| *H* | *Region* | *k* | *Z* | *x* | *y* | *z* |
| R | Amygdala | 8 | 2.52 | 22 | -6 | -20 |
| L | Amygdala | 8 | 2.54 | -22 | -8 | -20 |
| **White Matter** | | | | | | |
| *H* | *Metric and Region* | *k* | *Z* | *x* | *y* | *z* |
| R | FA - Corpus Callosum (body) | 2137 | 3.39 | 10 | -26 | 26 |
| - | FA - Corpus Callosum (splenium) | 29 | 3.38 | 0 | -35 | 11 |
| L | FA - Cerebral Peduncle | 28 | 2.78 | -14 | -15 | -13 |
| R | FA - Cerebral Peduncle | 24 | 2.89 | 16 | -12 | -10 |
| L | AD - Corpus Callosum (body) | 2003 | 4.93 | -2 | 6 | 24 |
| R | AD - Anterior Thalamic Radiation | 414 | 7.27 | 11 | -32 | 13 |
| R | AD - Corpus Callosum (genu) | 90 | 3.78 | 3 | 26 | 0 |
| L | AD - Forceps Major | 23 | 2.64 | -26 | -58 | 14 |
| R | AD - Cerebral Peduncle | 20 | 3.61 | 19 | -18 | -7 |
| L | AD - Cerebral Peduncle | 19 | 3.22 | -12 | -13 | -15 |
| R | AD - Corpus Callosum (genu) | 11 | 2.81 | 8 | 32 | 4 |
| L | AD - Forceps Major | 10 | 8.37 | -6 | -41 | 3 |
| L | AD - Cerebral Peduncle | 10 | 3.02 | -17 | -17 | -10 |
| R | AD - Cerebral Peduncle | 10 | 3.10 | 10 | -14 | -17 |
| R | MD - Anterior Thalamic Radiation | 94 | 5.21 | 6 | -5 | 7 |
| L | MD - Corpus Callosum (body) | 93 | 9.27 | -2 | 6 | 24 |
| R | MD - Anterior Thalamic Radiation | 44 | 5.08 | 12 | 6 | 4 |
| L | MD - Anterior Thalamic Radiation | 39 | 6.52 | -4 | -5 | 8 |
| L | MD - Anterior Thalamic Radiation | 34 | 5.11 | -12 | 5 | 5 |
| L | MD - Forceps Major | 28 | 17.1 | -6 | -41 | 3 |
| R | MD - Inferior Fronto-occipital Fasciculus | 28 | 5.34 | 24 | -14 | -9 |
| L | MD - Cingulum (hippocampus) | 25 | 4.98 | -25 | -54 | 4 |
| R | MD - Anterior Thalamic Radiation | 19 | 7.25 | 13 | 13 | -5 |
| L | MD - Corticospinal Tract | 17 | 18.50 | -6 | -9 | -24 |
| R | MD - Inferior Longitudinal Fasciculus | 17 | 5.50 | 39 | -31 | -18 |
| L | MD - Anterior Thalamic Radiation | 15 | 7.59 | -2 | -26 | -4 |
| L | MD - Forceps Major | 14 | 5.02 | -26 | -59 | 4 |
| R | MD - Superior Longitudinal Fasciculus | 14 | 3.78 | 34 | -17 | 59 |
| L | MD - Cerebral Peduncle | 12 | 3.18 | -12 | -25 | -19 |
| L | MD - Fornix | 12 | 5.02 | -30 | -15 | -15 |
| - | MD - Corpus Callosum (genu) | 10 | 4.28 | 0 | 25 | 0 |
| R | MD - Forceps Minor | 10 | 5.42 | 10 | 31 | -12 |
| R | RD - Anterior Thalamic Radiation | 92 | 4.09 | 6 | -5 | 7 |
| L | RD - Forceps Major | 27 | 13.2 | -6 | -41 | 3 |
| L | RD - Corpus Callosum (body) | 26 | 6.43 | -2 | 6 | 24 |
| L | RD - Anterior Thalamic Radiation | 17 | 6.44 | -2 | -26 | -4 |
| L | RD - Corticospinal Tract | 17 | 13.9 | -5 | -11 | -23 |
| R | RD - Inferior Longitudinal Fasciculus | 16 | 4.66 | 39 | -31 | -18 |
| L | RD - Anterior Thalamic Radiation | 16 | 3.47 | -14 | 0 | 6 |
| R | RD - Forceps Minor | 13 | 10.8 | 5 | -41 | 6 |
| L | RD - Forceps Major | 13 | 4.08 | -26 | -59 | 4 |
| L | RD - Inferior Fronto-occipital Fasciculus | 11 | 3.67 | -10 | -90 | -5 |
| R | RD - Forceps Minor | 10 | 4.69 | 10 | 31 | -12 |

The table lists the localization of local maxima of significant clusters encompassed in IC8. We reported clusters with size ⪰ 5 voxels for grey matter, and ⪰10 voxels for white matter microstructural features. The anatomical localization of significant clusters was performed with the JHU White-Matter Tractography Atlas and the JHU ICBM-DTI-81 White-Matter Labels (Hua et al., 2008; Wakana et al., 2007) for DTI features, while the SPM Anatomy toolbox (Eickhoff et al., 2005) was used to localize grey matter features. *H=hemisphere; k=cluster size (i.e., number of voxels); Z=z-scores; x, y, z=MNI coordinates; FA=Fractional Anisotropy; AD=Axial Diffusivity; MD=Mean Diffusivity; RD=Radial Diffusivity.*

**Table S4. Localization and coordinates of IC11**

| **Grey Matter** | | | | | | |
| --- | --- | --- | --- | --- | --- | --- |
| *H* | *Region* | *k* | *Z* | *x* | *y* | *z* |
| R | Rolandic Operculum | 2018 | 5.50 | 46 | -28 | 18 |
| R | Insula |  | 4.83 | 40 | 16 | 2 |
| L | Heschls Gyrus | 1153 | 5.10 | -46 | -16 | 10 |
| L | Superior Temporal Gyrus |  | 4.42 | -52 | -32 | 16 |
| R | Infirior Temporal Gyrus | 992 | 7.10 | 48 | -56 | -26 |
| L | Cerebelum (Crus 1) | 633 | 4.46 | -18 | -87 | -22 |
| R | Middle Temporal Gyrus | 593 | 6.12 | 45 | -52 | 20 |
| L | Superior Medial Gyrus | 555 | 4.36 | 0 | 42 | 26 |
| L | ACC |  | 3.53 | 3 | 48 | 8 |
| L | Cerebelum (VI) | 481 | 3.44 | -9 | -63 | -9 |
| L | Lingual Gyrus |  | 3.38 | -3 | -63 | 0 |
| L | Calcarine Gyrus |  | 3.01 | -8 | -60 | 6 |
| L | Middle Temporal Gyrus | 433 | 7.77 | -42 | -52 | 16 |
| R | Angular Gyrus | 314 | 4.70 | 32 | -51 | 42 |
| R | Lingual Gyrus | 295 | 4.07 | 10 | -60 | 15 |
| R | Calcarine Gyrus |  | 2.64 | 9 | -54 | 3 |
| L | Middle Occipital Gyrus | 219 | 5.62 | -32 | -62 | 36 |
| L | IFG (p. Triangularis) | 203 | 4.27 | -36 | 38 | 14 |
| R | Middle Frontal Gyrus | 147 | 3.89 | 30 | 15 | 46 |
| R | Middle Occipital Gyrus | 141 | 3.56 | 40 | -74 | 6 |
| L | Postcentral Gyrus | 133 | 4.45 | -54 | -20 | 27 |
| L | Cerebelum (VIII) | 133 | 3.18 | -21 | -69 | -54 |
| L | Hippocampus | 127 | 3.77 | -33 | -38 | -4 |
| L | Middle Frontal Gyrus | 109 | 4.23 | -38 | 14 | 39 |
| **White-Matter** | | | | | | |
| *H* | *Metric and Region* | *k* | *Z* | *x* | *y* | *z* |
| L | FA - Anterior Thalamic Radiation | 32 | 3.71 | -20 | -55 | 40 |
| L | FA - Inferior Longitudinal Fasciculus | 22 | 3.57 | -34 | -67 | 22 |
| L | FA - Corpus Callosum (body) | 20 | 3.02 | -16 | -14 | 35 |
| R | FA - Superior Longitudinal Fasciculus | 18 | 4.46 | 40 | -52 | 37 |
| R | FA - Inferior Fronto-occipital Fasciculus | 15 | 3.51 | 22 | -58 | 31 |
| L | FA - Superior Longitudinal Fasciculus | 15 | 3.76 | -34 | -42 | 45 |
| R | FA - Inferior Longitudinal Fasciculus | 12 | 3.51 | 34 | -66 | 20 |
| R | FA - Superior Corona Radiata | 12 | 3.21 | 18 | -1 | 37 |
| R | FA - Forceps Major | 11 | 3.13 | 20 | -80 | 1 |
| L | FA - Inferior Longitudinal Fasciculus | 10 | 4.33 | -17 | -84 | -5 |
| - | AD - Fornix | 82 | 7.47 | -1 | -9 | 16 |
| R | AD - Anterior Thalamic Radiation | 43 | 6.9 | 14 | -34 | 7 |
| L | AD - Anterior Thalamic Radiation | 30 | 6.72 | -17 | -34 | 9 |
| R | AD - Anterior Thalamic Radiation | 17 | 4.37 | 3 | -17 | -9 |
| L | AD - Anterior Thalamic Radiation | 13 | 8.24 | -11 | -30 | 6 |
| R | AD - Inferior Longitudinal Fasciculus | 13 | 4.24 | 50 | -52 | 10 |
| - | AD - Fornix | 12 | 6.27 | 0 | -1 | 13 |
| L | AD - Uncinate Fasciculus | 10 | 4.22 | -9 | 11 | -9 |
| - | MD - Fornix | 88 | 8.74 | -1 | -9 | 16 |
| R | MD - Anterior Thalamic Radiation | 49 | 8.26 | 14 | -34 | 7 |
| L | MD - Anterior Thalamic Radiation | 34 | 6.90 | -16 | -33 | 10 |
| R | MD - Anterior Thalamic Radiation | 21 | 4.92 | 11 | 9 | 0 |
| R | MD - Anterior Thalamic Radiation | 20 | 5.29 | 3 | -17 | -9 |
| - | MD - Corpus Callosum (body) | 15 | 4.63 | -1 | 7 | 23 |
| L | MD - Anterior Limb of Internal Capsule | 15 | 4.89 | -11 | 8 | -2 |
| R | MD - Inferior Longitudinal Fasciculus | 14 | 5.76 | 38 | -34 | -19 |
| L | MD - Forceps Major | 14 | 3.37 | -12 | -89 | 21 |
| R | MD - Superior Longitudinal Fasciculus | 12 | 4.67 | 31 | -46 | 56 |
| R | MD - Anterior Thalamic Radiation | 12 | 4.35 | 9 | -30 | -10 |
| R | MD - Anterior Thalamic Radiation | 11 | 3.37 | 2 | -30 | -14 |
| R | MD - Forceps Minor | 11 | 4.93 | 9 | 29 | -11 |
| R | MD - Inferior Longitudinal Fasciculus | 10 | 4.58 | 50 | -52 | -10 |
| L | MD - Uncinate Fasciculus | 10 | 5.22 | -10 | 12 | -10 |
| - | RD - Fornix | 84 | 8.21 | -1 | -9 | 16 |
| R | RD - Anterior Thalamic Radiation | 33 | 7.86 | 14 | -34 | 7 |
| L | RD - Anterior Thalamic Radiation | 32 | 6.04 | -16 | -33 | 10 |
| R | RD - Anterior Thalamic Radiation | 23 | 4.92 | 11 | 9 | 0 |
| R | RD - Anterior Thalamic Radiation | 20 | 5.06 | 3 | -17 | -9 |
| L | RD - Anterior Thalamic Radiation | 17 | 4.96 | -10 | 8 | 0 |
| R | RD - Inferior Longitudinal Fasciculus | 16 | 5.40 | 38 | -34 | -19 |
| - | RD - Corpus Callosum (body) | 16 | 4.56 | -1 | 7 | 23 |
| L | RD - Forceps Major | 13 | 3.72 | -8 | -90 | 20 |
| R | RD - Corticospinal Tract | 11 | 4.21 | 9 | -31 | -10 |
| R | RD - Anterior Thalamic Radiation | 10 | 3.29 | 2 | -30 | -14 |
| R | RD - Forceps Minor | 10 | 4.67 | 9 | 29 | -11 |

The table lists the localization of local maxima of significant clusters encompassed in IC11. We reported clusters with size ⪰ 80 voxels for grey matter, and ⪰10 voxels for white matter microstructural features. The anatomical localization of significant clusters was performed with the JHU White-Matter Tractography Atlas and the JHU ICBM-DTI-81 White-Matter Labels (Hua et al., 2008; Wakana et al., 2007) for DTI features, while the SPM Anatomy Toolbox (Eickhoff et al., 2005) was used to localize grey matter features. *H=hemisphere; k=cluster size (i.e., number of voxels); Z=z-scores; x, y, z=MNI coordinates: ACC=Anterior Cingulate Cortex; IFG= Inferior Frontal Gyrus; FA=Fractional Anisotropy; AD=Axial Diffusivity; MD=Mean Diffusivity; RD=Radial Diffusivity.*

**Figure S1. Correlation between IC6 and participants’ executive profile**


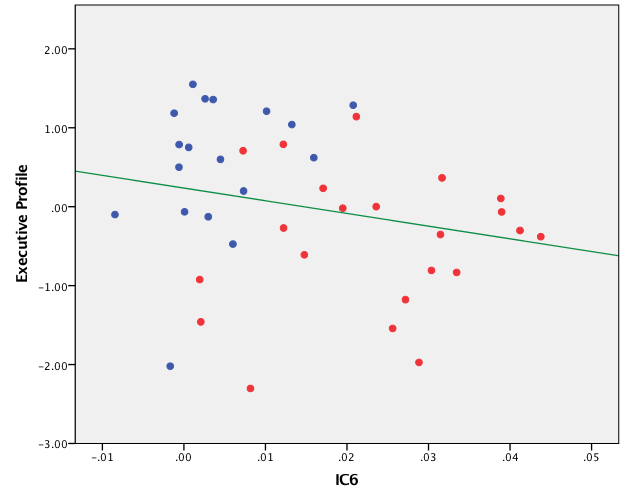


The scatterplot depicts the (non-significant) relationship (green line; *r=-0.23, p=0.159*) between IC6 mixing coefficients (x-axis) and the participants’ executive profile (i.e., proxy of basic executive performance as a result of a PCA on ENB-2 scores; y-axis). Red dots indicate AUD patients and blue dots indicate HC.

**Figure S2. Correlation between IC11 and participants’ executive profile**


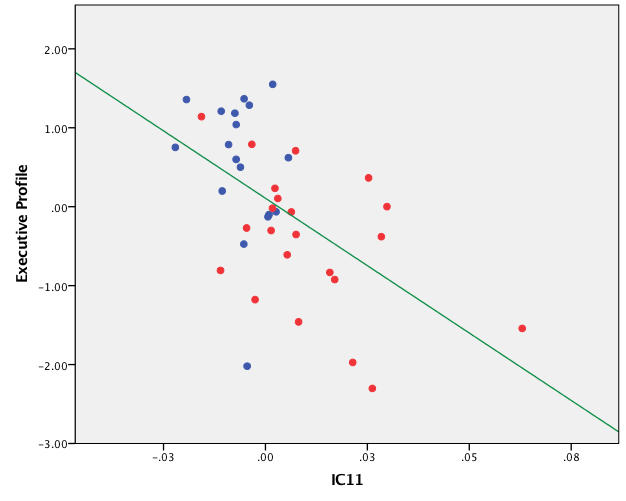


The scatterplot depicts the significant relationship (green line; *r=-0.54, p<0.001*) between IC11 mixing coefficients (x-axis) and the participants’ executive profile (i.e., proxy of basic executive performance as a result of a PCA on ENB-2 scores; y-axis). Red dots indicate AUD patients and blue dots indicate HC.

**Figure S3. Correlation between IC6 and lifetime duration of alcohol abuse**


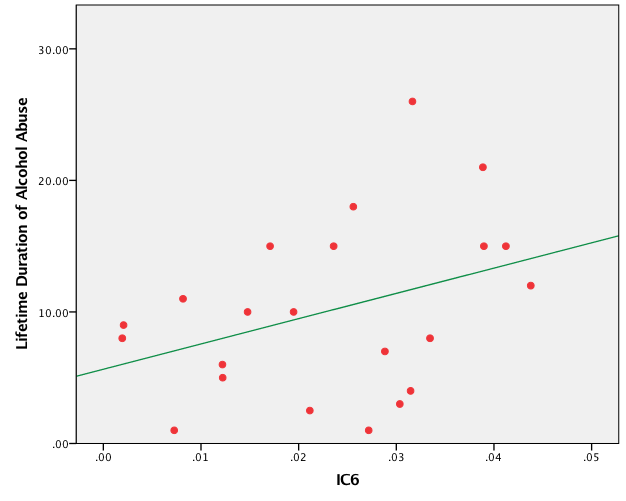


The scatterplot shows the (non-significant) relationship (*r=0.37, p=0.09*) between AUD patients’ IC6 mixing coefficients (x-axis) and the lifetime duration of alcohol abuse (years; y-axis).

**Figure S4. Correlation between IC11 and lifetime duration of alcohol abuse**


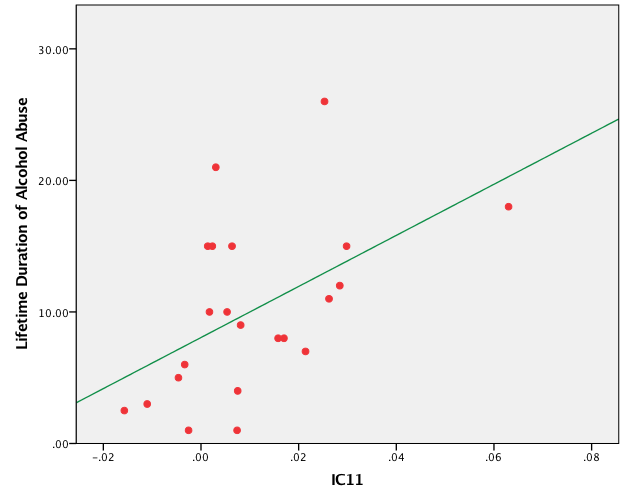


The scatterplot shows the significant relationship (*r=0.51, p=0.016*) between AUD patients’ IC11 mixing coefficients (x-axis) and the lifetime duration of alcohol abuse (years; y-axis).

**Figure S5. Correlation between IC6 and daily alcohol consumption**


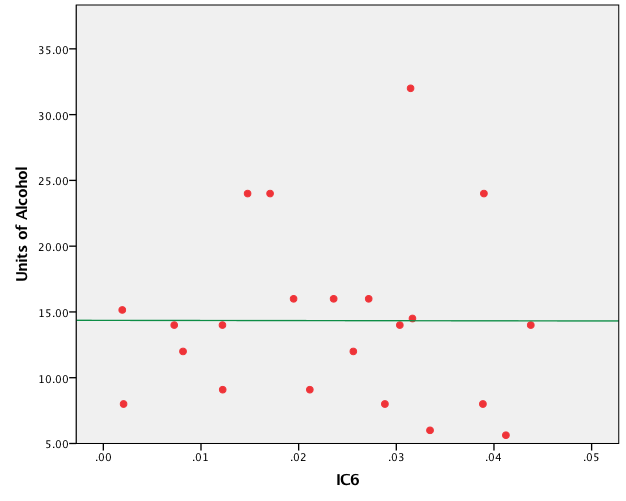


The scatterplot shows the (non-significant) relationship (*r=-0.002, p=0.994*) between AUD patients’ IC6 mixing coefficients (x-axis) and the daily alcohol consumption (Units of Alcohol; y-axis).

**Figure S6. Correlation between IC11 and daily alcohol consumption**


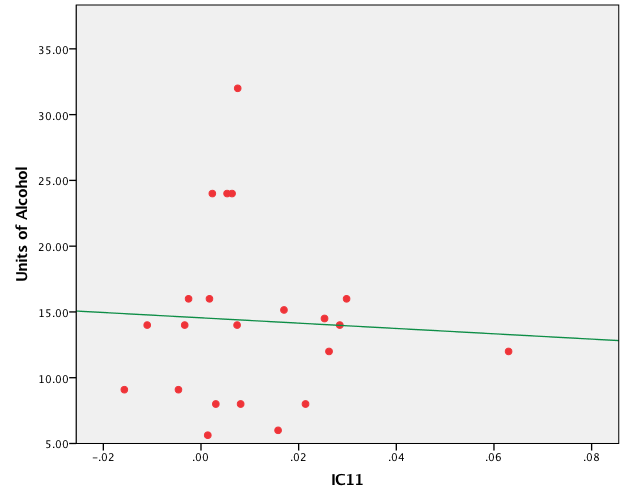


The scatterplot shows the (non-significant) relationship (*r=-0.05, p=0.499*) between AUD patients’ IC11 mixing coefficients (x-axis) and the daily alcohol consumption (Units of Alcohol; y-axis).

**References**

*Eickhoff S, Stephan KE, Mohlberg H, Grefkes C, Fink GR, Amunts K, et al. (2005). A new SPM toolbox for combining probabilistic cytoarchitectonic maps and functional imaging data. NeuroImage. doi:*[*10.1016/j.neuroimage.2004.12.034*](https://doi.org/10.1016/j.neuroimage.2004.12.034)

*Galandra, C., Basso, G., Manera, M., Crespi, C., Giorgi, I., Vittadini, G., et al. (2018b). Salience network structural integrity predicts executive impairment in alcohol use disorders. Sci. Rep. 8. doi:10.1038/s41598-018-32828-x.*

*Galandra, C., Basso, G., Manera, M., Crespi, C., Giorgi, I., Vittadini, G., et al. (2019). Abnormal fronto-striatal intrinsic connectivity reflects executive dysfunction in alcohol use disorders. Cortex 115. doi:10.1016/j.cortex.2019.01.004.*

*Hua, K., Zhang, J., Wakana, S., Jiang, H., Li, X., Reich, D. S., et al. (2008). Tract probability maps in stereotaxic spaces: Analyses of white matter anatomy and tract-specific quantification. Neuroimage. doi:10.1016/j.neuroimage.2007.07.053.*

*Mondini, S. (2011) Esame Neuropsicologico Breve 2: Una batteria di test per lo screening neuropsicologico. Cortina.*

*Wakana, S., Caprihan, A., Panzenboeck, M. M., Fallon, J. H., Perry, M., Gollub, R. L., et al. (2007). Reproducibility of quantitative tractography methods applied to cerebral white matter. Neuroimage. doi:10.1016/j.neuroimage.2007.02.049.*
